# Predictive model for 5-year mortality after breast cancer surgery in Taiwan residents

**DOI:** 10.1186/s40880-017-0192-9

**Published:** 2017-02-27

**Authors:** Su-Hsin Huang, Joon-Khim Loh, Jinn-Tsong Tsai, Ming-Feng Houg, Hon-Yi Shi

**Affiliations:** 1 Department of Nursing, Kaohsiung Municipal Hsiao-Kang Hospital, Kaohsiung Medical University, Kaohsiung, Taiwan China; 2 Department of Healthcare Administration and Medical Informatics, Kaohsiung Medical University, 100-Shih-Chun 1st Road, Kaohsiung, Taiwan China; 3 Divison of Neurosurgery, Department of Surgery, Kaohsiung Medical University Hospital, Kaohsiung, Taiwan China; 4 Department of Surgery, Kaohsiung Municipal Ta-Tung Hospital, Kaohsiung, Taiwan China; 5 Department of Computer Science, National Pingtung University, Pingtung, Taiwan China; 6 Division of General & Gastroenterological Surgery, Department of Surgery, Kaohsiung Municipal Hsiao-Kang Hospital, Kaohsiung, Taiwan China; 7 Cancer Center, Kaohsiung Medical University Hospital and Institute of Clinical Medicine, Kaohsiung Medical University, Kaohsiung, Taiwan China; 8 Department of Business Management, National Sun Yat-sen University, Kaohsiung, Taiwan China

**Keywords:** Breast cancer surgery, Artificial neural networks, Multiple logistic regression, Cox regression, 5-year mortality

## Abstract

**Background:**

Few studies of breast cancer surgery outcomes have used longitudinal data for more than 2 years. This study aimed to validate the use of the artificial neural network (ANN) model to predict the 5-year mortality of breast cancer patients after surgery and compare predictive accuracy between the ANN model, multiple logistic regression (MLR) model, and Cox regression model.

**Methods:**

This study compared the MLR, Cox, and ANN models based on clinical data of 3632 breast cancer patients who underwent surgery between 1996 and 2010. An estimation dataset was used to train the model, and a validation dataset was used to evaluate model performance. The sensitivity analysis was also used to assess the relative significance of input variables in the prediction model.

**Results:**

The ANN model significantly outperformed the MLR and Cox models in predicting 5-year mortality, with higher overall performance indices. The results indicated that the 5-year postoperative mortality of breast cancer patients was significantly associated with age, Charlson comorbidity index (CCI), chemotherapy, radiotherapy, hormone therapy, and breast cancer surgery volumes of hospital and surgeon (all *P* < 0.05). Breast cancer surgery volume of surgeon was the most influential (sensitive) variable affecting 5-year mortality, followed by breast cancer surgery volume of hospital, age, and CCI.

**Conclusions:**

Compared with the conventional MLR and Cox models, the ANN model was more accurate in predicting 5-year mortality of breast cancer patients who underwent surgery. The mortality predictors identified in this study can also be used to educate candidates for breast cancer surgery with respect to the course of recovery and health outcomes.

## Background

Breast cancer prevention is now a global health concern and is no longer limited to Western countries [1–3]. A 2011 report released from the Department of Health, Taiwan, China indicated that breast cancer in women was the fourth most common cause of cancer-related deaths among Taiwan women and the mortality was increasing each year [4]. Therefore, exploring the factors affecting the 5-year mortality after breast cancer surgery is imperative.

Few studies have compared the artificial neural network (ANN), multiple logistic regression (MLR), and Cox regression prediction models in terms of internal validity (reproducibility), which is an essential performance metric [5, 6]. However, variable predictive models are insufficient to reliably predict the long-term survival of patients after surgery.

The ANN model used in the present study was a standard feed-forward, back-propagation neural network with three layers: an input layer, a hidden layer, and an output layer. A multilayer perceptron (MLP) network is an emerging tool for designing special classes of layered, feed-forward networks [7]. The input layer comprises source neurons, and the output layer comprises outcome neurons; these two layers connect the network to the outside world. Additionally, an MLP network typically has one or more additional layers of neurons referred to as hidden neurons because they are not directly accessible. The hidden neurons extract important features contained in the input data.

An MLP network is typically trained using a back-propagation algorithm with forward and backward phases [7]. The back-propagation learning algorithm is easily implemented, and its linear complexity in the synaptic weights of the network makes it computationally efficient. For optimal learning efficiency, the neurons are typically activated with both anti-symmetric functions (e.g., hyperbolic tangent function) and non-symmetric functions (e.g., logistic function). The following cross-validation technique is used to optimize the time when an MLP network training session “stops.” An estimation dataset is used for training the model, and a validation dataset is used for evaluating model performance. The neural network is subsequently optimized using the training dataset. Finally, a separate testing dataset is used to determine when training should stop to mitigate over-fitting. The training cycle is repeated until the testing error no longer decreases [8, 9].

Although substantially improved outcome prediction models have been developed for many surgical procedures in recent years, studies of breast cancer outcome prediction models have had major shortcomings [10, 11]. For example, few studies have used longitudinal data for more than 2 years. Moreover, no breast cancer outcome prediction studies have considered group differences in factors such as age, gender, and non-surgical treatment. The present study used the ANN, MLR, and Cox models to identify the most influential factors for prediction of breast cancer surgery outcomes. The accuracy of various predictive models was also compared and analyzed using a global sensitivity analysis to assess the relative weights of significant predictors. The predictive simulations performed in the present study are expected to improve healthcare policies in Taiwan, China and the development of decision-supporting systems. Therefore, the primary aim of the present study was to validate the use of the ANN model for predicting 5-year mortality of breast cancer patients after surgery. The secondary aim was to compare predictive capability between the ANN, MLR, and Cox models.

## Patients and methods

### Study design and study population

In the present study, we applied a longitudinal research design based on a retrospective cohort study of patients who had undergone breast cancer surgery between January 1, 1996 and December 31, 2010 in Taiwan, China. The inclusion criteria were patients older than 18 years, who had received breast cancer surgery, and who were identified by database searches using ICD-9-CM 174.× diagnosis codes 174.0–174.9 and procedure codes 85.20–23, 85.33–36, 85.4×, 85.5×, 85.6×, 85.7×, 85.8×, and 85.95.

### Data collection

The present study analyzed data obtained from “the Bureau of National Health Insurance (BNHI)” in Taiwan, China. The BNHI database provided detailed administrative data regarding healthcare services, including outpatient visits, hospitalizations, and prescriptions, and has become extremely comprehensive [12]. The Longitudinal Health Insurance Database for year 2005 was established using a random sample with one million beneficiaries of all residents aged ≥18 years enrolled in the “National Health Insurance” program. The data source in this retrospective study was “the National Health Insurance Research Database” in Taiwan, China.

### Ethical considerations

The present study, which solely analyzed aggregate secondary data without identifying specific patients, was exempt from full review by the internal review board of Kaohsiung Medical University Hospital. Nevertheless, the study protocol still conformed to the ethical standards established with the 1964 Declaration of Helsinki, which waived the requirement for written or verbal patient consent in data linkage studies.

### Potential confounders

The research variables were categorized into patient and hospital characteristics based on the research covariates. The patient characteristics comprised age, comorbidity (circulatory system comorbidity and genitourinary system comorbidity), and treatment methods (chemotherapy, radiotherapy, and hormone therapy). Breast cancer surgery type was categorized as breast-conserving surgery, modified radical mastectomy, and mastectomy with reconstruction. Comorbidities identified according to ICD-9-CM codes for primary and secondary diagnoses were used to calculate Charlson comorbidity index (CCI) [13]. The hospital characteristics were surgery volumes of hospital and surgeon, and hospital level. For each hospital or surgeon, the surgery volume was defined as the number of breast cancer surgeries performed by the respective hospital or surgeon each year. Hospital level was recorded as medical center (>500 beds), regional hospital (301–500 beds), or district hospital (<300 beds) according to accreditation by the Taiwan Joint Commission on Hospital Accreditation.

The database was randomly separated into three datasets for training, testing, and external validation in a 7:2:1 ratio. In a probabilistic view of neural networks, this randomization can be viewed as a form of statistical sampling, such as Monte Carlo sampling. Once the optimization algorithm reached a certain level of precision, the stability and generalizability of the results obtained with a given ANN should be investigated using a jack-knife validation [14].

### Statistical analysis

The unit of analysis in the present study was the individual patient who underwent breast cancer surgery. The primary analytical methods were descriptive and inferential statistical analyses. The descriptive analyses had two objectives: (1) to describe the distribution of continuous variables using mean ± standard deviation (SD) and median in interquartile range (IQR); and (2) to describe the distribution of categorical variables using the number of total samples (N) and percentage (%). The inferential analysis comprised univariate and multivariate analyses using the ANN, MLR, and Cox models. The independent variables were age, CCI, surgery type, circulatory system comorbidity, genitourinary system comorbidity, chemotherapy, radiotherapy, hormone therapy, hospital level, surgery volumes of hospital and surgeon, and the dependent variable was the 5-year mortality of breast cancer patients after surgery. The discriminatory power of the models was also analyzed using the area under the receiver operating characteristic curves (AUC). Here, discriminatory power refers to the ability of a model to distinguish individuals who died from those who survived. A perfectly discriminatory model would assign a higher probability of death to patients who died than to patients who survived. For categorical variables, an overall test was applied to calculate the global *P* value, ensuring that the assumption of proportional hazards was not violated, and to identify any time-varying covariates.

The sensitivity analysis was performed to assess the importance of variables in the prediction models. The training process was simplified by introducing key variables and excluding all unnecessary variables. The sensitivity analysis was also performed to assess the relative significance of input variables in the prediction model and to rank the variables according to the order of importance. The sensitivity of the input variables against the output variables was expressed as the ratio of the network error (sum of squared residuals). A variable sensitivity ratio (VSR) of 1 or lower indicates that the variable diminishes network performance and should be removed.

The SPSS Version 20.0 statistical software (IBM SPSS Inc., Chicago, IL, USA) was used for statistical analyses.

## Results

### Patient selection

**Fig. 1:**
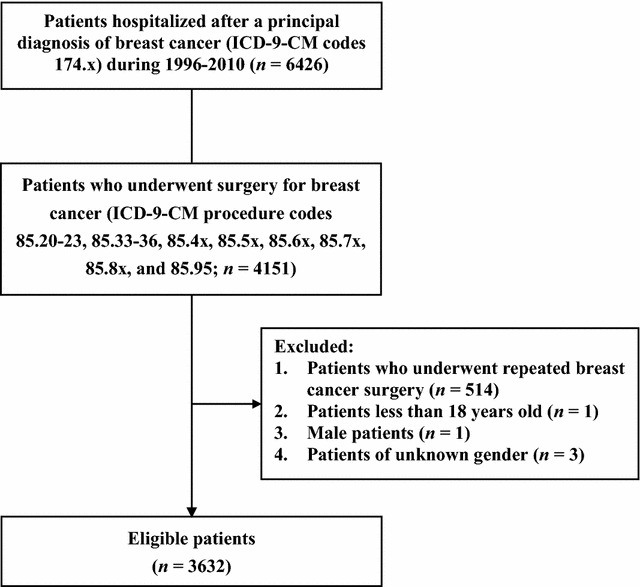
Flowchart of the study procedure

In total, 4151 patients underwent surgery for breast cancer during the study period. Among them, 519 patients were excluded, and 3632 were included for subsequent analyses (Fig. 1). The overall database was randomly divided into a model training dataset of 2543 cases, a model testing dataset of 726 cases, and a model validation dataset of 363 cases. The jack-knife method confirmed that the correlation between the classification probabilities of the prediction and the jack-knife validation was *R* = 0.93, suggesting a good stability of the results.

### Study characteristics

Table 1 shows the clinical and hospitalization characteristics of the patients analyzed in the present study. The median age of the patients was 52 years (IQR, 24–87 years). The median CCI was 1 (IQR, 0–1), and the occurrence rates of circulatory system and genitourinary system comorbidities were 7.8% and 6.5%. The median breast cancer surgery volume was 57 (IQR, 14–68) for hospitals and 14 (IQR, 4–29) for surgeons. Chemotherapy, radiotherapy, and hormone therapy were received by 41.8%, 11.0%, and 71.4% of the patients, respectively.

**Table 1:** Clinical and hospitalization characteristics of the 3632 selected patients who underwent surgery for breast cancer

Variable	Number of patients (%)
Breast cancer surgery type	
Breast-conserving surgery	1110 (30.6)
Modified radical mastectomy	2248 (61.9)
Mastectomy with reconstruction	274 (7.5)
Circulatory system comorbidity	
No	3347 (92.2)
Yes	285 (7.8)
Genitourinary system comorbidity	
No	3396 (93.5)
Yes	236 (6.5)
Chemotherapy	
No	2114 (58.2)
Yes	1518 (41.8)
Radiotherapy	
No	3233 (89.0)
Yes	399 (11.0)
Hormone therapy	
No	1037 (28.6)
Yes	2595 (71.4)
Hospital level	
Medical center	2109 (58.1)
Regional hospital	1341 (36.9)
District hospital	182 (5.0)
5-year outcome after surgery	
Death	502 (13.8)
Survival	3130 (86.2)

Table 2 shows the coefficients for 5-year mortality of the training dataset in the MLR model. The multivariate analysis results indicated that the 5-year mortality of breast cancer patients was significantly associated with age, CCI, chemotherapy, radiotherapy, hormone therapy, and breast cancer surgery volumes of hospital and surgeon (global *P* < 0.05). These significant variables were therefore included into these three prediction models.

**Table 2:** The analysis of the relationship between effective predictors and 5-year mortality of the 2543 breast cancer patients using the multiple logistic regression (MLR) model

Variable	Univariate analysis		Multivariate analysis	
	OR (95% CI)	*P* value	OR (95% CI)	*P* value
Age	1.02 (1.02–1.03)	0.001	1.03 (1.03–1.04)	0.001
Charlson comorbidity index	1.18 (1.14–1.22)	0.001	1.15 (1.11–1.20)	0.001
Circulatory system comorbidity				
Yes vs. no	1.08 (0.77–1.53)	0.641	0.89 (0.62–1.28)	0.543
Genitourinary system comorbidity				
Yes vs. no	0.76 (0.50–1.15)	0.198	0.84 (0.54–1.30)	0.441
Breast cancer surgery type				
MRM vs. BCS	1.04 (0.97–1.12)	0.247	1.03 (0.95–1.12)	0.448
MRM + TRAM vs. BCS	1.02 (0.98–1.06)	0.475	1.01 (0.98–1.04)	0.790
Chemotherapy				
Yes vs. no	1.57 (1.30–1.90)	0.001	1.92 (1.55–2.38)	0.001
Radiotherapy				
Yes vs. no	1.46 (1.11–1.92)	0.006	1.52 (1.13–2.05)	0.006
Hormone therapy				
Yes vs. no	0.74 (0.59–0.92)	0.006	0.79 (0.68–0.90)	0.006
Hospital level				
Medical center vs. district hospital	0.98 (0.94–1.02)	0.276	0.98 (0.95–1.02)	0.161
Regional hospital vs. district hospital	0.94 (0.86–1.03)	0.149	0.95 (0.89–1.02)	0.092
Surgery volume of hospital	0.94 (0.92–0.96)	0.001	0.95 (0.92–0.98)	0.004
Surgery volume of surgeon	0.93 (0.91–0.96)	<0.001	0.93 (0.90–0.97)	<0.001

*OR* odds ratio, *95*% *CI* 95% confidence interval, *MRM* modified radical mastectomy, *BCS* breast-conserving surgery, *TRAM* transverse rectus abdominis myocutaneous flap reconstruction. Surgery volume of hospital/surgeon was defined as the percentage of breast cancer surgeries among the total surgeries performed by the respective hospital or surgeon during the study period

### Comparisons between these three models

**Fig. 2:**
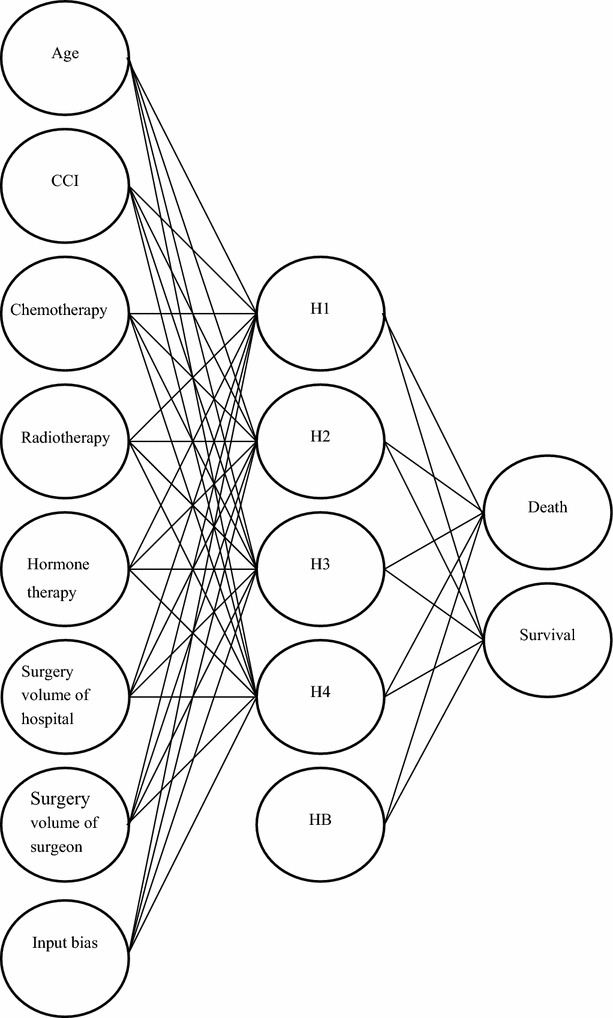
Schematic representation of the artificial neural network (ANN) model. This model consists of 7 input neurons [age, Charlson comorbidity index (CCI), chemotherapy, radiotherapy, hormone therapy, surgery volumes of hospital and surgeon], 1 bias neuron in the input layer, 4 neurons in a single hidden layer (H1-4), 1 bias neuron in the hidden layer (HB), and 2 output neurons (death and survival) representing the 5-year outcome of breast cancer patients after surgery. Surgery volume of hospital/surgeon was defined as the percentage of breast cancer surgeries among the total surgeries performed by the respective hospital or surgeon during the study period

The difference in the 5-year postoperative mortality of breast cancer patients between the training and testing datasets was not significant (data not shown). Therefore, samples from these two datasets could be compared to enhance the reliability of the validation results. The ANN model was also used to obtain the 3-layer network and the relative weights of neurons for the prediction of the 5-year mortality. The MLP network includes 7 input neurons, 1 bias neuron in the input layer, 4 hidden neurons, 1 bias neuron in the hidden layer, and 2 output neurons (Fig. 2).

Comparisons of samples in the training, testing, and validation datasets showed that the ANN model significantly outperformed the MLR and COX models in terms of sensitivity, specificity, positive predictive value, negative predictive value, accuracy, and AUC (Table 3).

**Table 3:** Comparison of performance indices of the artificial neural network (ANN), MLR, and Cox regression models for predicting 5-year postoperative mortality of breast cancer patients

Dataset	Sensitivity (%)	Specificity (%)	PPV (%)	NPV (%)	Accuracy (%)	AUC (%)
Training dataset (*n* = 2543)						
ANN model	86.88	89.09	65.24	87.91	87.43	72.86
MLR model	83.72	87.73	64.80	83.57	86.55	52.42
Cox model	86.37	88.06	60.24	85.48	84.53	65.13
Testing dataset (*n* = 726)						
ANN model	88.00	89.04	77.52	87.64	88.50	71.67
MLR model	83.00	84.36	73.35	87.02	86.09	51.93
Cox model	86.43	87.57	74.99	87.10	86.45	65.87
Validation dataset (*n* = 363)						
ANN model	85.66	88.71	53.42	86.89	88.82	70.76
MLR model	83.77	87.35	50.96	86.01	85.40	52.44
Cox model	84.61	85.43	52.02	86.60	86.91	64.79

*PPV* positive predictive value, *NPV* negative predictive value, *AUC* area under receiver operating characteristic curve

### Significant predictors in the ANN model

The training dataset was also used to calculate the VSRs for the MLP network. The global sensitivity analysis showed that the most sensitive variable for predicting 5-year mortality was breast cancer surgery volume of surgeon (VSR = 1.16), followed by breast cancer surgery volume of hospital (VSR = 1.14), age (VSR = 1.08), and CCI (VSR = 1.06). All VSR values exceeded 1, indicating that the network performed better when all variables were considered.

## Discussion

Performance indices were used to compare the three models in terms of accuracy in predicting breast cancer patient mortality. Overall, the mortality predicted using the ANN model was considerably more accurate than those predicted using the MLR and Cox models. When using actual outcome data for a performance comparison based on a simple outcome measure such as mortality, the ANN model clearly outperformed the MLR and Cox models constructed using the same limited number of clinical inputs.

Compared with reports that used data from a single medical center, the present registry study based on the data from BNHI, Taiwan, China provides a better overview of the current practice of breast cancer surgery. Unlike other single-center serial studies, the data from the present study and previous registry studies [15–17] provide an overview of the practices in large populations, avoiding referral bias or bias reflecting the practices of individual surgeons or institutions.

In recent years, studies have consistently shown that the ANN prediction model was superior to the MLR and Cox models [18–20]. Statistical analyses have also confirmed the advantages and preferred characteristics of the ANN model. The high fault tolerance of the ANN model also facilitates the appropriate and accurate processing of incomplete or noise-added inputs [13]. Moreover, highly correlated and non-normally distributed data can be used to construct linear and non-linear ANN models with broad applications in large-scale medical databases. In the medical field, the ANN model is commonly used for clinical research, especially for prognosis prediction [18–20]. Comparisons of various models in the present study suggested that the ANN predictive model was superior for expanding predictor variables to facilitate the systematic analysis of various diseases, for evaluating the effectiveness of research methods, and for comprehensively predicting mortality. The established predictive model can also be extended to other cancer types.

We conducted a global sensitivity analysis to assess the weights of significant predictors affecting the 5-year postoperative mortality of breast cancer patients. We found that surgery volume of surgeon was the most important predictor of 5-year mortality of breast cancer patients, consistent with the results of other reports [21, 22] showing that surgeons with high surgery volumes consistently achieve superior outcomes of breast cancer surgery than surgeons with low surgery volumes. Therefore, the treatment strategies made by these surgeons should be carefully analyzed and emulated. If the 5-year mortality is considered a benchmark, then breast cancer surgery volume of surgeon, a major predictor of postoperative outcomes, is crucial. Clearly, the outcomes of surgical procedures depend not only on clinical management but also on the skill and experience of individual surgeons. Moreover, surgeons with high surgery volumes in hospitals with high surgery volumes are most likely to achieve good patient outcomes because they are well supported by highly skilled interdisciplinary care teams [21].

Breast cancer surgery volumes of surgeon and hospital are the selection criteria typically considered by individuals seeking healthcare services. Patients also tend to seek treatment at large medical centers and by renowned, authoritative physicians [21, 22]. The results of the present study indicated that the surgery volumes of surgeon and hospital were negatively correlated with the 5-year mortality of breast cancer patients. That is, compared with their counterparts with low surgery volumes, hospitals and surgeons with high surgery volumes achieved higher patient survival rates. This finding provides a reference for healthcare institutions when assisting surgeons in cultivating relevant surgical experience.

The positive correlation observed between breast cancer mortality and age at onset requires further study. Strategies for improving screening rates to achieve early detection and treatment should also be investigated and compared. These data would provide a valuable reference for screening and for integrating therapeutic methods in predictive models for medical decision making.

Patients who undergo surgery for breast cancer are often burdened by cancer-related comorbidities that increase the risk of poor surgical outcomes, such as long hospitalization, high mortality, and high treatment costs [18–22]. In the present study, the statistical data for postoperative outcomes also revealed that 5-year mortality increased with CCI.

This long-term follow-up study analyzed patient data obtained from a database in Taiwan, China. In addition to enhancing the variance analysis of the correlation between treatment and survival, predictive models also have many applications in clinical care. The methods developed in the present study can also be used at healthcare institutions to evaluate the effectiveness of screening methods for early detection and treatment. Because of its accuracy in predicting breast cancer mortality, the proposed ANN model can be used to provide further data supporting the consensus view regarding the importance of breast cancer screening and the need for prompt and appropriate action. Beyond breast cancer, broader applications of this method can also facilitate the formulation and promotion of healthcare policies and the development of decision-supporting systems in Taiwan, China, which would ultimately enhance the health of all citizens. Additional studies are needed to determine the true clinical relevance of the ANN model and to clarify whether clinicians can effectively use the model to predict prognosis and optimize the surgical management for patients.

The present study has several limitations inherent in any large database analysis. First, the clinical picture obtained in the analysis of claims data is not as precise as that obtained in the analysis of a prospective clinical trial data, reflecting potential errors in the coding of primary diagnoses and surgical modalities. Second, complications associated with breast cancer surgical procedures were not assessed, limiting the validity of the comparison. Third, the specific focus on 5-year mortality as the endpoint of this prediction tool may limit the overall clinical use of the ANN model to a small subset of patients who have a high likelihood of death within 5 years. Fourth, only three models were used to predict 5-year survival after breast cancer surgery. Finally, other outcomes, such as patient-reported quality of life, were not compared because the relevant data were not included in the database. However, considering the robust magnitude and the statistical significance of the effects in the present study, these limitations are unlikely to compromise the validity of the results.

## Conclusions

Compared with the conventional MLR and Cox models, the ANN model in the present study was more accurate in predicting 5-year mortality of patients after breast cancer surgery and showed higher overall performance indices. The predictors analyzed in the present study could be addressed in preoperative and postoperative health care consultations to educate candidates for breast cancer surgery in the expected course of recovery and health outcomes. The international academic community has commended the researchers in Taiwan, China for the novel findings obtained by database studies. However, these studies can be further improved by including additional clinical variables, which could yield additional research findings and increase precision. Such findings can therefore provide a vital and indicative basis for promoting healthcare policies in Taiwan, China.

## References

[1] Chen W, Zheng R, Zeng H, Zhang S (2015). The updated incidences and mortalities of major cancers in China, 2011. Chin J Cancer.

[2] Chen WQ, Zheng RS, Zhang SW, Zeng HM, Zou XN (2014). The incidences and mortalities of major cancers in China, 2010. Chin J Cancer.

[3] Khan SA (2015). ASCO update on breast cancer, 2015. Surg Oncol.

[4] Chen BK, Yang CY (2013). Temporal trend analysis of avoidable mortality in Taiwan, 1971–2008: overall progress, with areas for further medical or public health investment. BMC Public Health.

[5] Zheng MH, Seto WK, Shi KQ, Wong DK, Fung J, Hung IF (2014). Artificial neural network accurately predicts hepatitis B surface antigen seroclearance. PLoS ONE.

[6] Çelik G, Baykan ÖK, Kara Y, Tireli H (2014). Predicting 10-day mortality in patients with strokes using neural networks and multivariate statistical methods. J Stroke Cerebrovasc Dis.

[7] Rumelhart DE, Hinton GE, Williams RJ, Rumelhart DE, McCleland JL (1986). Learning internal representations by error propagation. Parallel distributed processing: explorations in the microstructure of cognition.

[8] Haykin S (1999). Neural networks: a comprehensive foundation.

[9] Sandberg IW, Lo JT, Fancourt CL, Principe JC, Katagiri S, Haykin S (2001). Nonlinear dynamical systems: feedforward neural network perspectives.

[10] Chen C, Huang YB, Liu XO, Gao Y, Dai HJ, Song FJ (2014). Active and passive smoking with breast cancer risk for Chinese females: a systematic review and meta-analysis. Chin J Cancer.

[11] Tvedskov TF, Meretoja TJ, Jensen MB, Leidenius M, Kroman N (2014). Cross-validation of three predictive tools for non-sentinel node metastases in breast cancer patients with micrometastases or isolated tumor cells in the sentinel node. Eur J Surg Oncol.

[12] Chen YC, Wu JC, Haschler I, Majeed A, Chen TJ, Wetter T (2011). Academic impact of a public electronic health database: bibliometric analysis of studies using the general practice research database. PLoS ONE.

[13] D’Hoore W, Sicotte C, Tilquin C (1993). Risk adjustment in outcome assessment: the Charlson comorbidity index. Methods Inf Med.

[14] Huang KZ, Xiong XK, Zhang CM, Lai YY, Zou CN, Zhang GY (2014). Enhancement predicting accuracy for elastin-like polypeptides temperature transition by back propagation neural network. Protein Pept Lett.

[15] Hofvind S, Holen Å, Aas T, Roman M, Sebuødegård S, Akslen LA (2015). Women treated with breast conserving surgery do better than those with mastectomy independent of detection mode, prognostic and predictive tumor characteristics. Eur J Surg Oncol.

[16] Beek MA, Gobardhan PD, Klompenhouwer EG, Rutten HJ, Voogd AC, Luiten EJ (2015). Axillary reverse mapping (ARM) in clinically node positive breast cancer patients. Eur J Surg Oncol.

[17] Zhou HB, Liu SY, Lei L, Chen ZW, Peng J, Yang YZ (2015). Spatio-temporal analysis of female breast cancer incidence in Shenzhen, 2007–2012. Chin J Cancer.

[18] Iwase T, Yamamoto N, Ichihara H, Togawa T, Nagashima T, Miyazaki M (2014). The relationship between skeletal-related events and bone scan index for the treatment of bone metastasis with breast cancer patients. Medicine.

[19] Lu TC, Yu GR, Juang JC (2013). Quantum-based algorithm for optimizing artificial neural networks. IEEE Trans Neural Netw Learn Syst.

[20] Westreich D, Lessler J, Funk MJ (2010). Propensity score estimation: neural networks, support vector machines, decision trees (CART), and meta-classifiers as alternatives to logistic regression. J Clin Epidemiol.

[21] Shi HY, Hwang SL, Lee IC, Chen IT, Lee KT, Lin C (2014). Trends and outcome predictors after traumatic brain injury surgery: a nationwide population-based study in Taiwan. J Neurosurg.

[22] Lu CC, Chiu CC, Wang JJ, Chiu YH, Shi HY (2014). Volume-outcome associations after major hepatectomy for hepatocellular carcinoma: a nationwide Taiwan study. J Gastrointest Surg.

